# Supplementary material for: Bring dying at home: What facilitates and hinders home-based end-of-life care for people living with dementia?—A systematic review and meta-ethnography protocol
Source: PLoS One. 2024 Dec 30;19(12):e0316446. doi: 10.1371/journal.pone.0316446 (PMC11684621; doi:10.1371/journal.pone.0316446)
Supplement: S1 File — (DOCX) [file pone.0316446.s001.docx]

**MEDLINE Search Strategy**

1 exp Dementia/

2 dementia.tw.

3 Alzheimer*.tw.

4 exp Cognitive impairment/

5 Cognitive impairment.tw.

6 1 or 2 or 3 or 4 or 5

7 People.tw.

8 Person.tw.

9 Patient*.tw.

10 Older adult*.tw.

11 Individual*.tw.

12 Caregiver*.tw.

13 Family caregiver*.tw.

14 Care provider*.tw.

15 Health personnel*.tw.

16 Health worker*.tw.

17 Health professional*.tw.

18 Nurs*.tw.

19 Physician*.tw.

20 Hospice worker*.tw.

21 Social worker*.tw.

22 Stakeholder*.tw.

23 7 or 8 or 9 or 10 or 11 or 12 or 13 or 14 or 15 or 16 or 17 or 18 or 19 or 20 or 21 or 22

24 End of life care.tw.

25 exp end-of-life care/

26 exp palliative care/

27 palliative care.tw.

28 exp Hospice/

29 Hospice.tw.

30 exp Terminal care/

31 terminal care.tw.

32 exp dying/

33 dying.tw.

34 exp death/

35 death.tw.

36 die*.tw.

37 24 or 25 or 26 or 27 or 28 or 29 or 30 or 31 or 32 or 33 or 34 or 35 or 36

38 exp Home/

39 home.tw.

40 homes.tw.

41 House*.tw.

42 Residence.tw.

43 Domic*.tw.

44 Dwelling*.tw.

45 38 or 39 or 40 or 41 or 42 or 43 or 44

46 (Challeng* or Barrier* or Obstacle* or Hinder* or Enabl* or Block* or Deter* or Problem* or Difficult* or Stop* or Facilitat* or Allow* or Support* or Prevent* or Experience* or Perspectiv* or View* or Perception* or "Coping strateg*").tw.

47 ("Qualitative research" or "Qualitative study" or Phenomenolog* or "Case stud*" or "Narrative analys*" or "Focus group*" or "Semi structured interview*" or "Grounded theory" or "Mixed stud*").tw.

48 6 and 23 and 37 and 45 and 46 and 47
