# Supplementary material for: Bring dying at home: What facilitates and hinders home-based end-of-life care for people living with dementia?—A systematic review and meta-ethnography protocol
Source: PLoS One. 2024 Dec 30;19(12):e0316446. doi: 10.1371/journal.pone.0316446 (PMC11684621; doi:10.1371/journal.pone.0316446)
Supplement: S3 File — (DOCX) [file pone.0316446.s003.docx]

# Data extraction tool

| **Author, year** | **Country** | **Participants (n)** | **Aim** | **Data Collection** | **Data**  **Analysis** | **Main Results** | **Data Extractor** | **Date of Data Extraction** |
| --- | --- | --- | --- | --- | --- | --- | --- | --- |
